# Real-time ultrasound for umbilical venous catheter insertion in neonates- a systematic review and meta-analysis

**DOI:** 10.1186/s13089-025-00406-8

**Published:** 2025-01-13

**Authors:** Rajendra Prasad Anne, Emine A. Rahiman, Abhishek Somashekara Aradhya

**Affiliations:** 1 Department of Neonatology, Kasturba Medical College Manipal, Manipal Academy of Higher Education (MAHE), Manipal, Karnataka India; 2 Department of Pediatric Oncology, Kasturba Medical College Manipal, Manipal Academy of Higher Education (MAHE), Manipal, Karnataka India; 3 Ovum Women and Child Speciality Hospitals, Bengaluru, Hoskote, Karnataka India

**Keywords:** Malposition, Procedure duration, Sepsis, Mortality, Certainty of evidence

## Abstract

**Objective:**

There has been an increase in real-time ultrasonography use in central venous catheterisation. This systematic review and meta-analysis aimed to assess the role of real-time ultrasound use in umbilical venous catheterisation in neonates.

**Data sources:**

PubMed, Embase, Web of Science and Cochrane Library were searched on July 11, 2024. We followed the Cochrane Handbook for Systematic Reviews of Interventions (for study conduct), GRADE methodology (for certainty of evidence), and PRISMA guidelines (for reporting).

**Study selection:**

All randomised controlled trials/RCTs and non-randomised studies of interventions/NRSIs comparing real-time ultrasound with the conventional technique of umbilical venous catheterisation were included.

**Data extraction:**

The outcomes of interest were malposition rates, procedure duration, mortality, sepsis, and cost. Data extraction and quality assessment were done in duplicate.

**Data synthesis:**

Six studies (three RCTs and three NRSI), including 863 participants, were included. Data were analysed separately for RCTs and NRSIs. The RCTs were at low risk of bias, but NRSIs were at moderate to serious risk. The pooled estimates from RCTs showed a decrease in malposition rates (2 studies, 165 participants, risk ratio/RR 0.45, 95% confidence interval/CI 0.23, 0.90) and procedure duration (3 studies, 196 participants, mean difference −6.1 min, 95% CI −8.4, −3.8 min) with real-time ultrasound use. There was no reduction in sepsis. Mortality was not reported. The certainty of evidence was low for malposition rates and procedure duration. The data from NRSIs showed a reduction in malposition rates (3 studies, 667 participants, risk ratio/RR 0.10, 95% confidence interval/CI 0.07, 0.14) without an impact on procedure duration and sepsis. However, these findings did not improve the evidence.

**Conclusions:**

Low certainty evidence suggests that using real-time ultrasound for umbilical venous catheterisation reduces malposition rates. There is a clinically insignificant reduction in procedure duration. There is no sufficient data to come to a conclusion on the critical outcomes of sepsis and mortality.

*PROSPERO registration number*: CRD42024567895.

**Supplementary Information:**

The online version contains supplementary material available at 10.1186/s13089-025-00406-8.

## Introduction

Achieving quick and reliable vascular access is vital in managing critically ill neonates. While peripheral venous access suffices mostly, central venous access is required for the reliable delivery of vasoactive drugs and parenteral nutrition. An umbilical venous catheter (UVC) is the most common central venous catheter used in critically ill neonates and preterm neonates below 32 weeks gestational age. The procedure of UVC placement is blind, with the depth of insertion calculated using various formulae, including Shukla’s [1], Dunn’s [2], etc. While the procedure is quick and requires minimal personnel training, the catheter tip is often malpositioned. Malposition rates accounted for about 42% of adverse events related to UVC insertion in neonates [3]. Malposed catheters need to be repositioned, increasing the handling of the vascular access and exposure to radiographs. In some cases, repositioning may not be successful, requiring UVC removal. Inadvertent use of malpositioned lines, especially with a tip in the liver, can have disastrous complications [4].

Several measures have been proposed to improve the UVC malposition rates. These include but are not limited to using a double catheter technique, positioning the infant in the right lateral position, manual liver mobilisation, and using real-time ultrasound for tip location [5]. There is increasing data on utilising ultrasonography (US) for UVC insertion [6], confirmation of tip position [7], migration assessment [8], and confirmation of catheter-related adverse events. Ultrasonography is increasingly used in intensive care settings (neonatal, pediatric and adult) for central venous access. A recent meta-analysis included eight studies assessing the role of the US in peripherally inserted central catheters (PICC) in neonates [9]. Compared to X-rays, the US had a comparable sensitivity of 95.2% (95% CI 91.9%, 97.4%) and a lower specificity of 71.4% (95% CI 59.4%, 81.6%). In children, using the real-time US for central venous catheter insertion increased the likelihood of successful placement (likelihood ratio: 1.32; 95% CI 1.10, 1.58) and decreased the mean number of attempts (mean difference: −1.26 attempts; 95% CI -−1.71, −0.81) [10].

A recent systematic review and meta-analysis noted that ultrasound with saline contrast is superior to conventional anteroposterior X-rays for confirming UVC tip position in neonates [7]. There were no published meta-analyses on real-time or point-of-care ultrasound (POCUS) for UVC insertion. With this background, we performed a systematic review and meta-analysis comparing real-time ultrasound-guided umbilical venous catheterisation with the conventional blind technique to improve catheter malposition rates in neonates.

## Materials and methods

The protocol was prospectively registered with PROSPERO (CRD42024567895) and can be accessed at https://www.crd.york.ac.uk/ prospero/display_record.php?RecordID = 567895. We adhered to the methods of the Cochrane Handbook for Systematic Reviews of Interventions [11]. We reported as per Preferred Reporting Items for Systematic Reviews and Meta-Analyses (PRISMA) and Meta-analysis of Observational Studies in Epidemiology (MOOSE) guidelines [12, 13].

We included all randomised controlled trials (RCTs) and non-randomized studies of interventions (NRSIs) in which real-time ultrasound for umbilical venous catheterisation was compared with the conventional blind technique to decrease catheter malposition rates. We decided to include NRSIs because of the small number of trials available. We considered the malposition rates to be the primary outcome. The secondary outcomes were a) mortality, b) sepsis, c) the procedure duration, d) repositioning rates, e) adverse events related to UVC placement (liver lesions, thromboembolism, etc.) and f) cost. Malposition was defined as an inappropriate location of the UVC tip using an X-ray or an ultrasound. On X-ray, the tip position is determined using the cardiac silhouette or vertebral body methods [14]. The tip location in the inferior vena cava—right atrium (IVC-RA) junction was considered optimal on ultrasound [15, 16]. Mortality was defined as death before discharge due to any cause. Sepsis attributable to the umbilical line was defined as features of infection with positive blood culture occurring between 24 h of UVC insertion and 24 h of UVC removal [17]. The procedure duration was assessed from the initiation of the procedure to completion, i.e., suture placement. The cost attributable to UVC catheterisation would include the cost of material (catheter, disposable items), personnel, and procedure (X-ray or ultrasound).

We searched MEDLINE (PubMed; 1966 to July 2024), EMBASE (1980 to July 2024), the Cochrane Library (1996 to July 2024), and Web of Science (1964 to July 2024) on 11 July 2024. The search strategy is shown in eTable1, Supplementary Digital Content. The reference lists of the included studies and published reviews were also searched to identify relevant trials. To identify ongoing trials, we searched ClinicalTrials.gov and the ISRCTN registry. In addition, we searched grey literature through Google Scholar and ResearchGate websites. Two reviewers (RPA and EAR) independently performed the title and abstract screening and full-text screening. Any disagreements were resolved by mutual discussion or involvement of the third reviewer (ASA). The data extraction from the included studies was performed by two reviewers (RPA and ASA) in a blinded manner. Any disagreements were resolved by mutual discussion or involvement of the third reviewer (EAR). We extracted the following data on the methodology- setting, study design, inclusion and exclusion criteria, details of blind technique (personnel, formula for depth of insertion) and the ultrasound-guided technique (experience and training of personnel, ultrasound machine and probe details) and co-interventions used. The outcome data (gestational age, birth weight, day of life, and outcome details) were recorded in an Excel sheet.

We assessed the risk of bias using the Cochrane Risk of Bias tool, version 2 (RoB2) for RCTs [18] and the Risk of Bias in Non-randomized Studies of Intervention (ROBINS-I) tool for NRSIs [19]. We assessed RCTs in the domains of random sequence generation, allocation concealment, the blinding of the participants and personnel, the blinding of the outcome assessment, selection of the reported result, and other possible sources of bias. For NRSIs, we assessed the risk of bias due to confounding, selection of participants, classification of interventions, departures from intended interventions, missing data, measurement of outcomes, and the selection of reported results.

### Statistical analysis

We decided to conduct a meta-analysis if at least two studies compared similar interventions and comparators and measured the outcome(s) similarly. The meta-analysis was performed separately for RCTs and NRSIs, given their distinct risk of bias. Heterogeneity was explored through consideration of the study populations (e.g. differences in gestational age and birth weight), interventions (e.g. different formulae for depth of insertion, expertise of personnel performing ultrasound), outcome definitions (e.g. tip position assessed by X-ray versus ultrasound) and in statistical terms, by the I2 statistic. The I2 statistic, with a level of > 50%, indicated moderate heterogeneity and I2 > 80% as significant heterogeneity. Given the similar nature of intervention across the studies, a fixed-effects model was used. The meta-analysis was performed using the Cochrane statistical package, RevMan 5.4 software. The effects of the intervention were expressed as risk ratio (RR) for dichotomous data and as mean difference (MD) for continuous data, with 95% confidence intervals (CI).

If permitted, sensitivity analyses were planned to compare overall estimates with estimates from studies at low risk of bias. The reasons for significant heterogeneity were planned to be analysed when I2 was > 50%. Subgroup analyses were planned for different birth weight groups (< 1000 g vs > 1000 g), gestational age groups (< 28 weeks vs > 28 weeks), and settings (high vs. low- and middle-income countries). The certainty of evidence was downgraded when the heterogeneity was unexplained. We planned to assess the publication bias using funnel plots if the number of studies was more than 10. The Cochrane Grading of Recommendations Assessment, Development, and Evaluation (GRADE) approach [20] was used to assess the level of evidence (LOE).

## Results

**Fig. 1:**
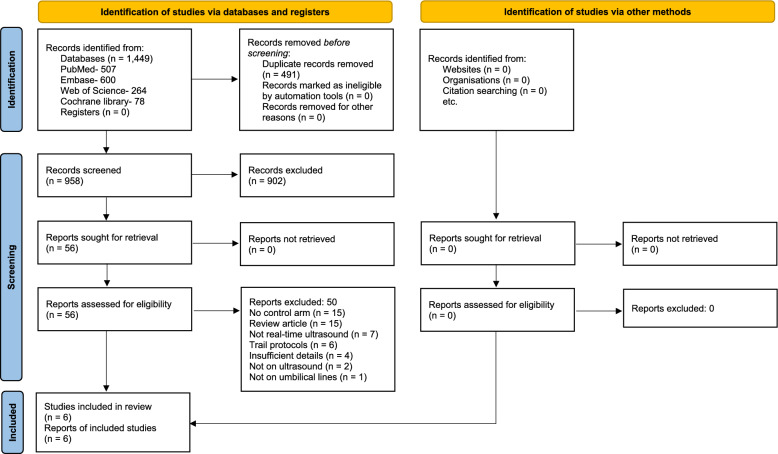
PRISMA flow diagram

Six studies, including 3 RCTs [21–23] and 3 NRSI [24–26], were included in the meta‐analysis. Four ongoing RCTs were identified [27–30]. The PRISMA flow diagram is shown in Fig. 1. The list of excluded studies is provided in eTable 2, Supplementary Digital Content. The six included studies provided data on 863 participants: 438 in the intervention group (ultrasound-guided UVC insertion) and 425 in the standard practice group. The characteristics of the included studies are shown in Table 1. Three studies were from high-income countries [23–25], one from upper-middle-income [26] and two from lower-middle-income countries [21, 22]. The characteristics of the participants are shown in Table 2. One author provided additional data on request [21].

**Table 1:** Characteristics of included studies

Author, year, study design	Standard blind procedure	Ultrasound guided insertion	Co-interventions
Fleming 2011 RCT	Personnel- Neonatal fellows, neonatal nurse practitioners and pediatric house staff Length- Left to practitioner Tip position- X-ray	Personnel: Neonatologist experienced in real-time USG or a resident under supervision Machine: 13 MHz Linear Probe, Vivid I, General Electric Healthcare Tip position- X-ray	USG group- Catheter manipulations were suggested when suboptimal catheter positioning; Viewed in real time until suitable position obtained
Kaur 2022 RCT	Personnel- Trained neonatal fellow Length- Shukla and Ferrara formula Tip position- X-ray	Personnel- Single investigator trained in point-of-care ultrasonography for neonates Machine- Sonosite Edge with a sector probe (4–8 Hz) Tip position- X-ray	USG group- Aberrant course into portal vein- gentle pressure was applied with the ultrasound probe to compress the hepatic portal venous sinuses, till the catheter tip reached the right position
Mishra 2024 RCT	Personnel- Not specified Length- predetermined formula (not specified) Tip position- X-ray	Personnel- Primary investigator (neonatologist) after 15 day training in radiology department Machine- Fujifilm Sonosite (M Turbo) ultrasound machine (Washington, USA), 4–8 MHz probe, 0.5 ml saline flush for better visualisation Tip position- X-ray	USG group- Pressure was given on the liver (upper abdomen) by the transducer to align the umbilical vein with the ductus venosus
Rossi 2022 Retrospective	Personnel- Not specified Length- Shukla and Ferrara formula Tip position- X-ray	Personnel- 3 consultant neonatologists trained under a radiologist Machine- 8 C convex probe (4–11 MHz) from a LOGIQe US machine (GEHealthcare^®^) Tip position- X-ray	None specified
Guzmán-de la Garza 2020 Retrospective	Personnel- Resident doctor, assisted by a nurse Length- Shukla and Ferrara formula Tip position- X-ray	Personnel- Resident doctor, assisted by a nurse. Another resident as ultrasound operator Machine- 7–18 MHz linear probe using US machine from Chison Medical Imaging Company Tip position- Ultrasound	None specified
D’Andrea 2024 Retrospective	Personnel- Consultant neonatologist, or a resident with sufficient training under supervision of neonatologist Length- Shukla formula Tip position- X-ray	Personnel- Consultant neonatologist, or a resident with sufficient training under supervision of neonatologist; Tip navigation by another operator experienced in ultrasound visualization Machine- S4–10 micro sectorial probe set to 7 MHz using the LOGIQ E9 Ultrasound Machine (GE Healthcare) Tip position- Ultrasound	USG group- facilitation maneuvers during insertion X-ray group- tip was corrected by some amount to achieve a central position if it was in the heart or a peripheral position if the it was in the prehepatic site

*RCT* randomised controlled trial, *USG* ultrasonography, *UVC* umbilical venous catheter

**Table 2:** Characteristics of participants

Author, year	Sample size	Male gender	Gestational age (weeks) Mean (SD) or Median [IQR]	Birth weight (grams) Mean (SD) or Median [IQR]	Age Mean (SD) or Median [IQR]
Fleming 2011	Intervention- 15 Control- 16	Not specified	31 + 4 (4 + 1) 30 + 2 (5 + 2)	1728 (1133) 1312 (917)	Not specified
Kaur 2022	Intervention- 26 Control- 27	19 (73%) 21 (78%)	33.4 (4.5) 32.5 (4.5)	1987 (903) 1741 (755)	2 [1, 2] days 1 [1, 2] days
Mishra 2024	Intervention- 58 Control- 54	28 (48%) 32 (59%)	33.7 (4.5) 33.5 (4.3)	1,672 [1,060–2,365] 1,445 [1,020–2,245]	5 [2–14] hrs 2 [2–12] hrs
Rossi 2022	Intervention- 38 Control- 54	Not specified	31.5 [30–32]	1,889 [1,643–2,135]	Not specified
Guzmán-de la Garza 2020	Intervention- 52 Control- 62	Not specified	33.6 (3.6) 33.4 (3.6)	1,893 (846) 1,859 (776)	Not specified
D’Andrea 2024	Intervention- 249 Control- 212	Not specified	32.9 (5.24)	1909 (1032)	Not specified

*SD* standard deviation, *IQR* interquartile range

The risk of bias assessment is summarised in Table 3. While the RCTs were of good quality, the NRSIs had a moderate to serious risk of bias, predominantly due to problems in analysis methods (no measures were taken to control for confounding domains) and outcome assessment (lack of blinding of outcome assessment, and different reference standards were used for tip confirmation in intervention and standard treatment arms).

**Table 3:** Risk of bias assessment

a. Randomised controlled trials						
Author, year	Randomization process	Deviations from intended interventions	Missing outcome data	Measurement of the outcome	Selection of the reported result	Overall Bias
Fleming, 2011	Low	Some concerns^a^	Low	Low	Some concerns^b^	Some concerns
Kaur, 2022	Low	Low	Low	Low	Low	Low
Mishra, 2024	Low	Some concerns^a^	Low	Low	Low	Some concerns

b. Non-randomised studies of intervention								
Author, year	Bias due to confounding	Bias inselection of participants in to the study	Bias in classification of interventions	Bias due to deviation from intended interventions	Bias due to missing data	Bias in measurement of outcomes	Bias in selection of the reported result	Overall bias
Rossi, 2022	Moderate^c^	Low	Low	Low	Low	Moderate^d^	Low	Moderate
Guzmán-de la Garza, 2020	Moderate^c^	Low	Low	Low	Low	Serious^d,e^	Low	Serious
D’Andrea, 2024	Moderate^c^	Low	Low	Low	Low	Serious^d,e^	Low	Serious

^a^Intention to treat analysis was not used

^b^A prespecified analysis plan or a published protocol was not found

^c^Did not use appropriate analysis methods to control confounding domains and time-varying confounding

^d^The outcome assessors were not blinded/ no information was provided on blinding

^e^The gold standard for confirming tip position differed in the intervention (ultrasound technique was used) and control (X-ray was used) groups

**Fig. 2:**
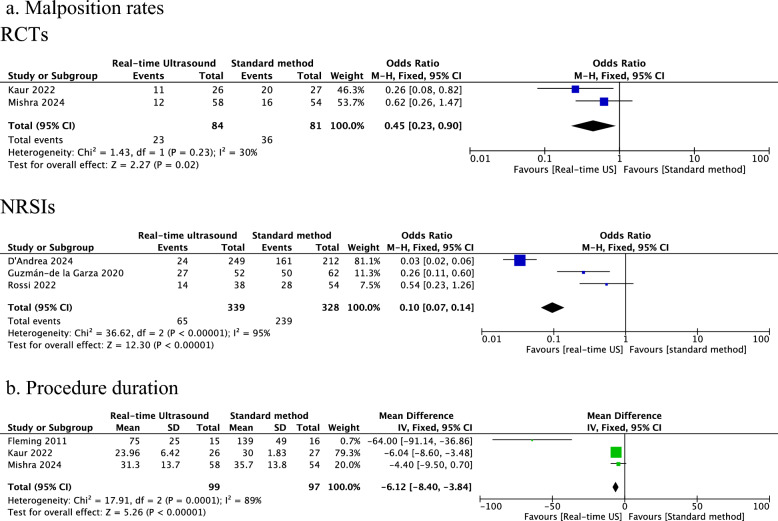
Forest plots. *RCTs* Randomised controlled trials, *NRSIs* Non-randomised studies of interventions, *M-H* Mantel–Haenszel method, *CI* confidence interval, *IV* Inverse variance, *US* Ultrasound

The results of the meta-analysis are shown in Table 4. The forest plots are shown in Fig. 2. On meta-analysis of RCTs, a statistically significant reduction was noted in the malposition rates (2 studies, 165 neonates; RR 0.45, 95% CI 0.23, 0.90) and the procedure duration (3 studies, 196 participants; MD −6.1 min, 95% CI −8.4 min, −3.8 min). Analysis of NRSIs also showed reduced malposition rates (3 studies, 667 neonates; RR 0.10, 95% CI 0.07, 0.14) but no decrease in procedure duration (Table 4). None of the studies reported the outcome of mortality. There was no significant reduction in sepsis incidence, both in RCTs (1 study, 112 neonates; RR 0.44; 95% CI 0.10, 1.84) and NRSIs (1 study, 114 neonates; RR 3.73; 95% CI 0.38, 37.03). Rossi et al. and Guzmán-de la Garza et al. reported the cost for X-rays but not the overall cost difference per our definition. Hence, we did not consider it for analysis.

**Table 4:** Meta-analysis

Outcome	Study type	Participants (studies)	Risk ratio (95% CI) Mean difference (95% CI)	Certainty of evidence
Malposition rates	RCTs	165 (2)	0.45 (0.23, 0.9)	LOW^a,b^
	NRSI	667 (3)	0.10 (0.07, 0.14)	VERY LOW^c,d^
Procedure duration	RCTs	196 (3)	−6.1 (−8.4, −3.8) minutes	LOW^d^
	NRSIs	114 (1)	−2 (−8.6, 4.6) minutes	VERY LOW^a,c,e^
Sepsis	RCTs	112 (1)	0.44 (0.10, 1.84)	LOW^a,e^
	NRSIs	114 (1)	3.73 (0.38, 37.03)	VERY LOW^a,c,e^

*CI* confidence interval, *RCT* randomised controlled trial, *NRSI* non-randomised studies of interventions

^a^Wide confidence interval (downgraded by 1 for imprecision)

^b^Risk of bias (downgraded by 1)

^c^Serious risk of bias (downgraded by 2)

^d^I2 of > 75% (downgraded by 2 for serious inconsistency)

^e^Data was from one single-centre study (downgraded by 1 for inconsistency)

We could not explore the reasons for heterogeneity further due to inadequate studies. We could not assess for publication bias using the funnel plots, as only three studies were available. We could not perform preplanned subgroup analyses. The certainty of evidence assessed using the GRADEpro software is shown in Table 4. The certainty of evidence varied from low to very low for the outcomes studied. Including data from the NRSIs did not improve the certainty of evidence.

## Discussion

In this systematic review and meta-analysis, we assessed the role of real-time ultrasound in umbilical venous catheterisation. Three RCTs and three NRSIs were included in the meta-analysis. We found low-certainty evidence that using real-time ultrasound decreases malposition rates and reduces the duration of the procedure. Very low-certainty evidence suggested no impact on neonatal sepsis rates. The outcomes of mortality and cost of care were not reported.

An anteroposterior X-ray is typically used to confirm the tip position. However, it has some disadvantages, including delay in initiating infusions awaiting the X-ray, exposure to ionising radiation, and logistic challenges in procuring the X-ray [31]. Recent studies have emphasised the role of ultrasonography in estimating the correct UVC tip location in various scenarios- to assess tip navigation during the procedure (real-time) [32], to confirm the tip position after placement (post-procedure) [7] and to assess the tip location while in use (migration assessment) [8]. In a recent meta-analysis, X-ray was shown to have a comparable sensitivity of about 90% (95% CI 71%, 97%) and a lower specificity of 82% (95% CI 53%, 95%) when compared to ultrasound or echocardiography, for confirming tip location [7]. The reason for higher accuracy with ultrasound could be the difficulty in capturing expiratory film with X-ray in neonates. As a result, the diaphragm could have a dynamic position, varying with the phase of respiration and lung expansion, especially in ventilated neonates. All these factors indicate that ultrasound can potentially replace X-ray in umbilical catheterisation.

In a meta-analysis assessing complications of UVC in over 14,000 neonates, malposition was the commonest adverse event, accounting for about 41.7% (95% CI 27.6%, 56.5%) of the adverse events (13.4% of all UVCs had an adverse event) [3]. Malposition results in the handling of the neonate (line repositioning or removal), the need for repeat X-rays to confirm the tip location and additional expenditure. Increased handling of central venous devices may increase the risk of infections and result in unwarranted morbidity. Hence, it is imperative to adopt evidence-based strategies to decrease malposition rates.

The reliability of the US depends on the operator's skill (knowledge of anatomical landmarks, skill in using the US, and experience) and the appropriateness of the equipment used (US machine resolution and type of probe chosen) [33]. If strict asepsis protocols are not adhered to, concern regarding the increased risk of sepsis is relevant. Such confounding factors highlight the need for structured training programmes to improve the operator's skills. A recent protocol suggested using small sectorial probes, 7–8 MHz, with a low subcostal longitudinal view for assessing tip navigation and a subcostal longitudinal view for assessing tip location [32].

Although we followed the Cochrane Handbook methodology, the study has a few limitations. The data was limited, and only a few studies with few participants were available. The sample size from high-quality studies (RCTs) is insufficient for the primary outcome of malposition. About 45% of neonates enrolled in the control arm had malposition in 2 studies that provided data in this meta-analysis [21, 22]. Suppose real-time US use was to reduce the malposition rates by about 25% (i.e., to approximately 34%). The total sample size required is 650, assuming equal group sizes to achieve a power of 80% for detecting a difference in proportions of −0.11 between the two groups (test-reference group) at a two-sided p-value of 0.05 [34]. The critical outcomes of mortality and sepsis were not adequately reported. All the studies were single-centre studies. Hence, there is a need for large multi-centric trials with adequate sample sizes to answer this research question. Such studies should evaluate and report the outcomes of sepsis related to UVC (i.e., central-line associated bloodstream infections/CLABSI) and complications specific to UVC placement (e.g., hepatic lesions and thrombosis), differences in the cost of care due to use of real-time ultrasound, in addition to the critical outcome of mortality.

## Conclusion

We conclude that low certainty evidence suggests a reduction in malposition rates and procedure duration with real-time ultrasonography during umbilical venous catheterisation. Further studies are required to assess the effect of real-time ultrasound on sepsis and mortality outcomes.

## Supplementary Information

Supplementary material 1.

## Data Availability

All the data used for the meta-analysis is presented in the tables, figures and the text. If further details are required, the corresponding author will provide on a reasonable request.

## Abbreviations

CI: Confidence interval
GRADE: Grading of recommendations, assessment, development and evaluation
IVC-RA junction: Inferior vena cave—right atrium junction
LOE: Level of evidence
MD: Mean difference
MHz: Mega hertz
MOOSE: Meta-analysis of observational studies in epidemiology
NRSI: Non-randomised study of intervention
PICC: Peripherally inserted central catheter
POCUS: Point-of-care ultrasound
PRISMA: Preferred reporting items for systematic reviews and meta-analyses
RCT: Randomised controlled trial
RoB-2: Risk of bias version 2
ROBINS-I: Risk of bias in non-randomised studies of intervention
US: Ultrasonography
UVC: Umbilical venous catheter
