# Supplementary material for: Real-time ultrasound for umbilical venous catheter insertion in neonates- a systematic review and meta-analysis
Source: Ultrasound J. 2025 Jan 13;17:4. doi: 10.1186/s13089-025-00406-8 (PMC11730037; doi:10.1186/s13089-025-00406-8)
Supplement: Supplementary file 1 — Supplementary material 1. [file 13089_2025_406_MOESM1_ESM.docx]

**Title:** Ultrasound guidance for umbilical catheterisation in neonates: a systematic review and meta-analysis

eTable 1: Search Strategy

| Database | Strategy | Limits |
| --- | --- | --- |
| PubMed | (neonate OR newborn OR infant OR preterm OR "low birth weight") AND (“umbilical catheter” OR “umbilical cannula” OR “umbilical vein” OR “umbilical venous” OR “umbilical artery” OR “umbilical arterial” OR “umbilical vessel” OR “umbilical vascular”) AND (insertion OR placement OR position OR access OR catheteri*ation) AND (ultrasound OR sonogram OR sonograph* OR sonolog* OR echocardiogra*) | None |
| Embase | (neonate OR newborn OR infant OR preterm OR ‘low birth weight’) AND (‘umbilical catheter’ OR ‘umbilical cannula’ OR ‘umbilical vein’ OR ‘umbilical venous’ OR ‘umbilical artery’ OR ‘umbilical arterial’ OR ‘umbilical vessel’ OR ‘umbilical vascular’) AND (insertion OR placement OR position OR access OR catheteri*ation) AND (ultrasound OR sonogram OR sonograph* OR sonolog* OR echocardiogra*) | None |
| Web of Science | ALL=((neonate OR newborn OR infant OR preterm OR low-birth-weight) AND (umbilical-catheter OR umbilical-cannula OR umbilical-vein OR umbilical-venous OR umbilical-artery OR umbilical-arterial OR umbilical-vessel OR umbilical-vascular) AND (insertion OR placement OR position OR access OR catheteri*ation) AND (ultrasound OR sonogram OR sonograph* OR sonolog* OR echocardiogra*)) | None |
| Cochrane | (neonate OR newborn OR infant OR preterm OR low birth weight) AND (umbilical catheter OR umbilical cannula OR umbilical vein OR umbilical venous OR umbilical artery OR umbilical arterial OR umbilical vessel OR umbilical vascular) AND (insertion OR placement OR position OR access OR catheteri*ation) AND (ultrasound OR sonogram OR sonograph* OR sonolog* OR echocardiogra*) in Title Abstract Keyword | Title, abstract and key words |

eTable 2: List of excluded studies

| **S No** | **Author, Year, Country** | **Tile** | **Reason for exclusion** |
| --- | --- | --- | --- |
|  | Pradhan JB, 2021, India | Role of Point of care Echocardiography during securing the umbilical venous catheterization in neonates - A randomized control trial | Trial protocol (Not yet recruiting) |
|  | Mishra P, 2023, India | Comparison of Ultrasound guided umbilical insertion with conventional method- A randomized control trial | Trial protocol (Study already published) |
|  | Kaur A, 2021, India | Ultrasound guided umbilical venous catheter insertion to reduce rate of catheter tip malposition in neonates | Trial protocol (Study already published) |
|  | Singh S, 2021, India | Ultrasound guided insertion of umbilical catheters in neonates  CTRI/2018/04/013126 | Trial protocol (Open to recruitment) |
|  | Kumar M, 2023, India | Study comparing how good an ultrasound scan guided long catheter insertion technique is in guiding the catheter to the correct position, when compared to the usual technique | Trial protocol (Not yet recruiting) |
|  | Ponin L, 2024, Thailand | Comparison of Point of Care Ultrasound (POCUS) and X-ray in confirmation the position of central line and endotracheal tube in the neonatal intensive care unit (NICU)  TCTR20230906002 | Trial protocol (Recruiting) |
|  | D’Andrea, 2024 | Real-Time Ultrasound Tip Location Reduces Malposition and Radiation Exposure during Umbilical Venous Catheter Placement in Neonates: A Retrospective, Observational Study | Different Gold Standard |
|  | Guzmán-de la Garza, 2020 | Cateterización venosa umbilical guiada por ultrasonografía: un análisis de coste-efectividad | Different Gold Standard |
|  | Ades, 2003 | Echocardiographic Evaluation of Umbilical Venous Catheter Placement | No control arm |
|  | Akar, 2020 | Determination of Accurate Position of Umbilical Venous Catheters in Premature Infants | No control arm |
|  | Ang, 2010 | Ultrasound as the imaging of choice in determining optimal umbilical venous catheter placement | No control arm |
|  | Barone, 2022 | Neo-ECHOTIP: A structured protocol for ultrasound-based tip navigation and tip location during placement of central venous access devices in neonates. | Review article |
|  | Collard 2018 | Seeing is believing: The value of ultrasound-guided umbilical catheter placement | Review article |
|  | D'Andrea 2022 | Real-Time Ultrasound Tip Location Reduces Malposition and Radiation Exposure during Umbilical Venous Catheter Placement in Neonates: A Retrospective, Observational Study. | Review article |
|  | DeWitt 2015 | Fluoroscopy-guided Umbilical Venous Catheter Placement in Infants with Congenital Heart Disease | Not on ultrasound |
|  | El-Maadawy 2015 | Role of Bedside Ultrasound in Determining the Position of Umbilical Venous Catheters | Not real-time ultrasound |
|  | Garg 1983 | Positioning of umbilical arterial catheters with ultrasound | No control arm |
|  | George 1982 | Umbilical vascular catheters: localization by two-dimensional echocardio/aortography. | No control arm |
|  | Greenberg 1995 | Placement of umbilical venous catheters with use of bedside real-time ultrasonography. | Inadequate details of intervention arm |
|  | Houston 1982 | Ultrasound positioning of umbilical arterial catheters. | No control arm |
|  | Karber 2017 | Optimal radiologic position of an umbilical venous catheter tip as determined by echocardiography in very low birth weight newborns. | Not real-time ultrasound |
|  | Kempley 1992 | Randomised trial of umbilical arterial catheter position: Doppler ultrasound findings. | Not real-time ultrasound |
|  | Kishigami 2020 | Ultrasound-Guided Umbilical Venous Catheter Insertion With Alignment of the Umbilical Vein and Ductus Venosus. | No control arm |
|  | Kochan 2021 | Point-of-Care Ultrasound to Confirm Umbilical Line Placement: Impact of a Simulation Enhanced Curriculum on Neonatal Intensive Care Unit Provider Competency. | No control arm |
|  | Kozyak 2022(a) | Real-Time Ultrasound Guidance for Umbilical Venous Cannulation in Neonates With Congenital Heart Disease. | No control arm |
|  | Kozyak 2022(b) | Real-time Ultrasound Guidance to Increase Success of Umbilical Venous Cannulation | No control arm |
|  | Meinen 2020 | Point-of-care ultrasound use in umbilical line placement: a review. | Review article |
|  | Mele 2020 | Neonatal Nurse Practitioner Use of Ultrasonography to Verify Umbilical Venous Catheter Placement in the Neonatal Intensive Care Unit. | No control arm |
|  | Michel 2012 | Comparison of ultrasound and X-ray in determining the position of umbilical venous catheters. | Not real-time ultrasound |
|  | Nguyen 2016 | Ultrasonography for Central Catheter Placement in the Neonatal Intensive Care Unit - A Review of Utility and Practicality | Review article |
|  | Pittiruti 2023 | A GAVeCeLT bundle for central venous catheterization in neonates and children: A prospective clinical study on 729 cases. | Not on umbilical lines |
|  | Pollaci 1989 | Insertion of umbilical arterial catheters with guidance by ultrasound. | Not real-time ultrasound |
|  | Polonio 2022 | Implementation of point of care ultrasound to assess umbilical venous catheter position in the neonatal intensive care unit | No control arm |
|  | Pulickal 2013 | Superiority of targeted neonatal echocardiography for umbilical venous catheter tip localization: accuracy of a clinician performance model. | Not real-time ultrasound |
|  | Rajaraman 2023 | Does the use of point of care ultrasound (POCUS) improve umbilical venous catheter (UVC) positioning in neonates? | Review article |
|  | Rosen 1970 | Umbilical Venous Catheterization in the Newborn: Identification of Correct Positioning | Not real-time ultrasound |
|  | Rubortone 2021 | Real-time ultrasound for tip location of umbilical venous catheter in neonates: a pre/post intervention study. | Data comparing US-guided insertion with blind technique not available |
|  | Saul 2015 | Ultrasound for central vascular catheter and endotracheal tube position in the NICU-a pilot study | No control arm |
|  | Saul 2016 | Sonography for Complete Evaluation of Neonatal Intensive Care Unit Central Support Devices: A Pilot Study. | No control arm |
|  | Seibert 1987 | Sonographic detection of neonatal umbilical-artery thrombosis: clinical correlation. | Not real-time ultrasound |
|  | Seigel 2020 | Use of clinician-performed ultrasound in the assessment of safe umbilical venous catheter tip placement. | Not real-time ultrasound |
|  | Shabeer 2020 | Bedside sonography performed by neonatology residents to confirm central vascular catheter position in neonates - A Prospective Diagnostic Evaluation study. | Not real-time ultrasound |
|  | Sharma 2019 | Role of ultrasound for central catheter tip localization in neonates: a review of the current evidence. | Review article |
|  | Simanovsky 2011 | Umbilical venous catheter position: evaluation by ultrasound. | Not real-time ultrasound |
|  | Sobczak 2021 | Ultrasound Monitoring of Umbilical Catheters in the Neonatal Intensive Care Unit—A Prospective Observational Study | Not real-time ultrasound |
|  | Thakur 2020 | Use of Point of Care Ultrasound for Confirming Central Line Tip Position in Neonates | Not real-time ultrasound |
|  | Torres 2023 | Steps to improve umbilical vein catheterization in neonatal care. | Data comparing US-guided insertion with blind technique not available |
|  | Tsui 2005 | Umbilical vein catheterization under electrocardiogram guidance. | Not on ultrasound |
|  | Wren 2024 | Affordable Implementation of a Point-of-Care-Ultrasound Program in a Large Tertiary NICU to Assess Umbilical Venous Catheter Tips and Aid Central Placement. | Data comparing US-guided insertion with blind technique not available |
|  | Xie 2023 | Point-of-care ultrasound for monitoring catheter tip location during umbilical vein catheterization in neonates: a prospective study. | No control arm |
